# Limited diagnostic possibilities for bloodstream infections with broad‐range methods: A promising PCR/electrospray ionization‐mass spectrometry platform is no longer available

**DOI:** 10.1002/mbo3.1007

**Published:** 2020-02-07

**Authors:** Jan Tkadlec, Eliska Bebrova, Jan Berousek, Tomas Vymazal, Jaroslava Adamkova, Vendula Martinkova, Claus Moser, Dragos Florea, Pavel Drevinek

**Affiliations:** ^1^ Department of Medical Microbiology 2nd Faculty of Medicine Charles University and Motol University Hospital Prague Czech Republic; ^2^ Department of Anaesthesiology and Intensive Care Medicine 2nd Faculty of Medicine Charles University and Motol University Hospital Prague Czech Republic; ^3^ 3rd Department of Surgery 1st Faculty of Medicine Charles University and Motol University Hospital Prague Czech Republic; ^4^ Department of Clinical Microbiology Rigshospitalet Copenhagen Denmark; ^5^ National Institute for Infectious Diseases University of Medicine and Pharmacy Carol Davila Bucharest Romania

**Keywords:** 16S PCR, bloodstream infections, broad‐range PCR, molecular diagnostics, PCR/ESI‐MS

## Abstract

Fast and accurate detection of causative agents of bloodstream infections remains a challenge of today's microbiology. We compared the performance of cutting‐edge technology based on polymerase chain reaction coupled with electrospray ionization‐mass spectrometry (PCR/ESI‐MS) with that of conventional broad‐range 16S rRNA PCR and blood culture to address the current diagnostic possibilities for bloodstream infections. Of 160 blood samples tested, PCR/ESI‐MS revealed clinically meaningful microbiological agents in 47 samples that were missed by conventional diagnostic approaches (29.4% of all analyzed samples). Notably, PCR/ESI‐MS shortened the time to positivity of the blood culture‐positive samples by an average of 34 hr. PCR/ESI‐MS technology substantially improved current diagnostic tools and represented an opportunity to make bloodstream infections diagnostics sensitive, accurate, and timely with a broad spectrum of microorganisms covered.

## Graphical abstract

By using the polymerase chain reaction coupled with electrospray ionization‐mass spectrometry (PCR/ESIMS) technology, we detected pathogenic agents in 29.4% (47 of 160) more samples than by using 16S rRNA PCR and/or blood culture. Thus, the PCR/ESI‐MS represented an opportunity to make the bloodstream infection diagnostics highly sensitive and accurate.

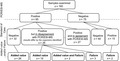

The use of molecular methods in medical microbiology improves the sensitivity of examinations, shortens the turnaround time (TAT), and allows the detection of uncultivable microorganisms (Opota, Jaton, & Greub, 2015). However, their application in the diagnostics of bloodstream infections (BSI) is troublesome due to an extremely low bacterial load and the presence of an excessive amount of human DNA in whole blood (Opota et al., 2015). Therefore, classical blood culture (BC) remains the cornerstone of BSI diagnostics; however, its TAT often exceeds 24 hr, and the sensitivity can be as low as 50% (Opota et al., 2015; Peker, Couto, Sinha, & Rossen, 2018). Thus, reliable and robust BSI diagnostics are an unmet need of today's medical microbiology.

In this respect, a commercial platform IRIDICA (Abbott Molecular, Des Plaines, IL, USA) that combines a set of PCRs including broad‐range PCRs with electrospray ionization‐mass spectrometry (hereafter, PCR/ESI‐MS) entered the European market in 2014, based on a very favorable outcome of a multicentre clinical trial (Vincent et al., 2015). The method is capable of detecting over 800 bacterial and *Candida* species associated with BSI and shortening the TAT by up to 6 hr (Bacconi et al., 2014). The width of detection, unprecedented for a commercial test, matches the conventional broad‐range PCR strategy (Tkadlec et al., 2019) and goes far beyond the possibilities of any pathogen‐specific PCR.

We aimed to evaluate the utility of the PCR/ESI‐MS system in a clinical setting by comparing its real‐life performance with conventional culture‐dependent (BC) and culture‐independent (16S ribosomal RNA PCR) broad‐range tests.

Within 12 months, we examined 166 blood samples from 137 patients (median age: 64 years; range 22–94 years; 72% males) who had been hospitalized with suspected BSI at intensive care units of Motol University Hospital in Prague.

A BC was performed by using BACTEC™ FX (Becton Dickinson) with one pair of aerobic and anaerobic BC bottles collected. These BC bottles were incubated for 5 days before being concluded as negative. Additional (mycotic) BC bottles were collected if patients were suspected of a mycotic infection, and these bottles were incubated for up to 14 days before being concluded as negative. Positive BC bottles were streaked out on solid media, and upon overnight culture, the microorganisms were identified by using a MALDI‐TOF mass spectrometer Biotyper v 3.1 (Bruker Daltonics). Two additional EDTA tubes were collected at the time of the BC blood draw. One tube was subjected to panbacterial/panfungal 16S/18S PCR assay (hereafter 16S‐PCR) using UMD‐SelectNA™ kit with Add‐On10 extension (Molzym) to process up to 10 ml of whole blood as previously described (Tkadlec et al., 2019). The other tube with 5 ml whole blood was stored frozen at −20°C for up to 3 months until being processed with the PCR/ESI‐MS system. The samples were run with the BAC BSI assay according to the manufacturer's instructions (Abbott Molecular).

We assessed every PCR/ESI‐MS result against the BC and 16S‐PCR results, which are collectively referred to as standard‐of‐care (SoC) tests. When a discrepancy between PCR/ESI‐MS and SoC occurred, the patient's medical records were carefully reviewed to determine whether the unique positivity of PCR/ESI‐MS meant a false negativity of the SoC tests and vice versa. Detection of typical contaminating microbiota by any of the tested methods was not regarded as a relevant finding unless it was supported by microbiological investigation of other materials from the same patient. The unique positivity obtained by PCR/ESI‐MS was considered of *added value* (Tkadlec et al., 2019) if a detected organism was found in another sample, collected close to the collection date of the sample for PCR/ESI‐MS, and/or the detected microorganism was known to be associated with the BSI. Conversely, to the *added value*, *failures* referred to the results in which the PCR/ESI‐MS missed relevant organism(s) that were detected by the SoC tests.

We obtained valid results from both PCR/ESI‐MS and SoC methods from 160 samples (Figure 1). More than half of the samples were either complete negative or double positive with agreement in terms of recovering the same microorganism(s). PCR/ESI‐MS as the only method detected the positivity or the presence of additional agent(s) in 58 samples. Out of them, 47 findings (i.e., 29.4% of all analyzed samples) were assessed to be of *added value*. Notably, 26 of the 47 samples were completely negative by the SoC tests, while 21 were positive by the SoC tests (5 with both BC and 16S‐PCR, 5 with BC only, and 11 with 16S‐PCR only); however, one or more clinically relevant microorganism(s) were left undetected (Appendix in Table A1). The *failure* of PCR/ESI‐MS was seen in seven cases.

**Figure 1:**
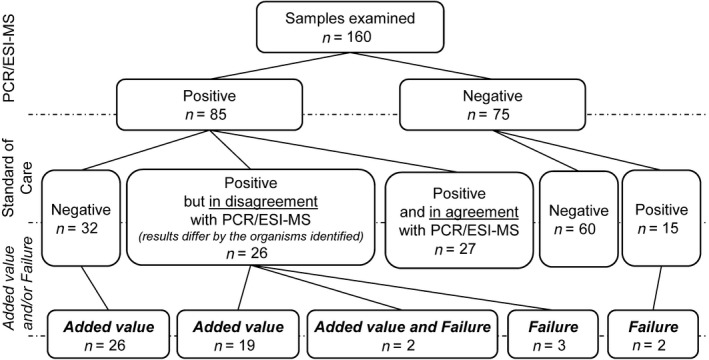
PCR/ESI‐MS results categorized by the level of agreement with the standard‐of‐care methods (i.e., blood culture and 16S‐PCR) and by its utility as defined by *added value* and/or *failure* (please note that two samples fulfilled criteria for both categories and are labeled as IDs 51 and 52 in Appendix in Table A1)

Time to culture positivity ranged from 4 to 116 hr for positive aerobic and anaerobic bottles and up to 271 hr for positive mycotic bottles. Time to culture positivity exceeded 24 hr in 16 of 27 BC samples, which had concordant results with the PCR/ESI‐MS. On average, the PCR/ESI‐MS result was delivered faster than that of BC by 34 hr if hands‐on and instrument run times were taken into consideration.

Reliable and timely diagnostics of BSI‐causing agents are critical for correct patient management. BC is the gold standard for BSI diagnostics, but due to its limitations, alternative detection systems are being sought. We tested the performance of PCR/ESI‐MS as a novel BSI diagnostic method and found that: (a) The method was able to detect clinically relevant causes of BSI in 30% of samples (that would be determined as negative by the SoC methods); and (b) PCR/ESI‐MS exhibited a considerably shorter time to positivity when compared to that of BC.

However, it is important to note that the majority of our patients were on antibiotic therapy at the time of sample collection and that the higher PCR/ESI‐MS positivity rate could be also attributed to the detection of free circulating DNA, not necessarily of viable microbial cells. However, positivity of PCR/ESI‐MS was already associated with increased mortality in patients with suspected sepsis, indicating the ability of the technology to correctly identify critically ill patients (O’Dwyer et al., 2017).

Despite these highly encouraging PCR/ESI‐MS results (Karrasch et al., 2018; Makristathis et al., 2018; Tassinari et al., 2018; Vincent et al., 2015), the technology was unexpectedly suspended in 2017 (Özenci, Patel, Ullberg, & Stralin, 2018). Thus, 16S‐PCR currently remains the only molecular genetic test with a panbacterial spectrum of detection that is applicable to blood. However, we previously demonstrated its low added value of 6.5% and high failure rate of 7.1% when blood samples from adults in intensive care units were checked in parallel with BC (Tkadlec et al., 2019). We believe that the discontinuation in the PCR/ESI‐MS technology development is a loss and an unfortunate step back in the quest for ideal BSI diagnostics, and we strongly encourage the diagnostic industry to develop methods and equipment that can provide similar advantages to the clinical service of PCR/ESI‐MS.

## CONFLICT OF INTEREST

The authors declare no conflict of interest.

## AUTHORS CONTRIBUTION

Jan Tkadlec: Conceptualization‐Supporting, Data curation‐Equal, Formal analysis‐Equal, Investigation‐Lead, Project administration‐Lead, Writing‐original draft‐Equal; Eliska Bebrova: Data curation‐Supporting, Formal analysis‐Supporting, Supervision‐Supporting, Validation‐Supporting; Jan Berousek: Methodology‐Supporting, Resources‐Supporting, Validation‐Supporting, Writing‐original draft‐Supporting; Tomas Vymazal: Formal analysis‐Supporting, Investigation‐Supporting, Methodology‐Supporting, Project administration‐Supporting, Resources‐Supporting, Supervision‐Supporting, Writing‐original draft‐Supporting; Jaroslava Adamkova: Methodology‐Supporting, Project administration‐Supporting, Validation‐Supporting; Vendula Martinkova: Formal analysis‐Supporting, Methodology‐Supporting, Project administration‐Supporting, Validation‐Supporting; Claus Moser: Methodology‐Supporting, Resources‐Supporting, Writing‐original draft‐Supporting; Dragos Florea: Methodology‐Supporting, Resources‐Supporting, Supervision‐Supporting, Writing‐original draft‐Supporting; Pavel Drevinek: Conceptualization‐Lead, Data curation‐Equal, Formal analysis‐Equal, Funding acquisition‐Lead, Investigation‐Supporting, Methodology‐Lead, Project administration‐Supporting, Resources‐Lead, Supervision‐Lead, Validation‐Lead, Writing‐original draft‐Equal.

## ETHICS STATEMENT

Study protocol had been reviewed and approved by the institutional Ethics Committee of the Motol University Hospital, Prague, Czech Republic (date of the application for approval: 17 July 2014). All subjects or their legal representatives provided written informed consent.

## DATA AVAILABILITY STATEMENT

The datasets generated and/or analyzed during the current study are not publicly available due to confidentiality reasons but are available from the corresponding author upon reasonable request.

## APPENDIX 1

**Table A1:** Individual microbiological findings of PCR/ESI‐MS and standard‐of‐care tests (i.e., blood culture and 16S‐PCR) in 52 samples with *added value* and/or *failure*

No.	PCR‐ESI/MS	16S‐PCR	BC
Added value			
1	***Escherichia coli***	Negative	Negative
2	***Enterobacter cloacae* complex**	Negative	Negative
3	***Escherichia coli*** *, Streptococcus pseudopneumoniae, Corynebacterium *sp.	CoNS	Negative
4	***Klebsiella pneumoniae***	Negative	Negative
5	***Leclercia adecarboxylata, Klebsiella pneumoniae*** *, Enterobacter cloacae* complex	*Enterobacter* sp., *Streptococcus oralis* group	*Enterobacter cloacae*
6	***Enterobacter cloacae* complex**, *Gemella haemolysans*, *Streptococcus* sp., *Candida glabrata/ C. albicans*	*Bacillus* sp.	Negative
7^a^	***Klebsiella pneumoniae***	Negative	Negative
8^a^	***Enterococcus faecium***	Negative	Negative
9	***Enterococcus faecalis*** *, Candida albicans*	Negative	*Candida albicans*
10	***Enterobacter cloacae* complex, *Escherichia coli***	Negative	Negative
11	***Fusobacterium necrophorum***	Negative	Negative
12	***Klebsiella variicola***	Negative	Negative
13^b^	***Enterococcus faecium***	pos. (with no ID retrieved)	Negative
14^b^	***Klebsiella oxytoca*** *, Staphylococcus haemolyticus*	CoNS, *Propionibacterium acnes*	*Staphylococcus haemolyticus*
15	***Odoribacter splanchnicus, Fusobacterium necrophorum, Escherichia coli, Enterobacter cloacae* complex**	Negative	Negative
16	***Salmonella bongori***	Negative	Negative
17	***Klebsiella pneumoniae, Escherichia coli, Morganella morganii***	Negative	Negative
18	***Klebsiella pneumoniae***	*Staphylococcus epidermidis*	Negative
19	***Klebsiella variicola*** *, Streptococcus pyogenes*	*Streptococcus pyogenes*	Negative
20^c^	***Serratia marcescens***	Negative	Negative
21^c^	***Serratia marcescens***	Negative	*Staphylococcus hominis*
22^c^	***Serratia marcescens***	Negative	Negative
23	***Burkholderia cepacia* complex, *Enterobacter cloacae* complex**	Negative	Negative
24	***Enterococcus faecium, Enterobacter cloaceae* complex**	CoNS	Negative
25	***Enterobacter cloacae* complex, *Escherichia coli***	*Bacillus* sp.	Negative
26	***Bacteroides uniformis*** *, Shigella boydii*	Negative	Negative
27	***Escherichia coli*** *, Nocardia *sp.*, Candida tropicalis*	Negative	*Nocardia* sp.
28	***Escherichia coli, Klebsiella pneumoniae, Bacteroides fragilis***	Negative	Negative
29	***Klebsiella pneumoniae***	Negative	Negative
30	***Pseudomonas aeruginosa***	Negative	Negative
31	***Escherichia coli,*** * Klebsiella pneumoniae*	*Klebsiella pneumoniae*	Negative
32	***Escherichia coli***	CoNS	Negative
33	***Streptococcus pyogenes***	negative	*Staphylococcus epidermidis*
34^d^	***Klebsiella *sp.** *, Propionibacterium acnes*	negative	negative
35^d^	***Klebsiella pneumoniae***	negative	negative
36	***Clostridium perfringens, Escherichia coli***	*Lactobacillus *sp.	negative
37	***Leclercia adecarboxylata, Klebsiella oxytoca, Enterobacter cloacae* complex**	*Bacillus *sp.*, Massilia *sp.	negative
38	***Candida parapsilosis*** *, Proteus mirabilis*	Negative	*Proteus mirabilis*
39	***Klebsiella pneumoniae***	Negative	negative
40	***Streptococcus pneumoniae***	Negative	negative
41	***Klebsiella pneumoniae***	Negative	negative
42	***Streptococcus agalactiae,*** * Staphylococcus aureus*	*Staphylococcus aureus*	*Staphylococcus aureus, Staphylococcus epidermidis*
43^e^	***Pseudomonas aeruginosa***	Negative	Negative
44^e^	***Pseudomonas aeruginosa***	Negative	Negative
45	***Escherichia coli***	Negative	Negative
Failure			
46	*Clostridium sticklandii*	***Streptococcus pyogenes***, ***Fusobacterium nucleatum***, *Gemmela* sp., *Parvimonas micra*, *Peptostreptococcus* sp.	***Streptococcus pyogenes***
47	*Escherichia coli*	*Escherichia coli*	***Klebsiella pneumoniae*** *, Escherichia coli*
48^b^	x	***Haemophilus parainfluenzae***	Negative
49	*Fusobacterium nucleatum*	***Parvimonas micra*** *, Fusobacterium nucleatum*	Negative
50	x	Negative	***Burkholderia cepacia* complex**
Added value and Failure			
51	***Streptococcus pneumoniae*,** Group G *Streptococcus*, *Pseudomonas aeruginosa, Propionibacterium acnes*	***Enterococcus* sp.,** CoNS	***Enterococcus faecalis*** *, Pseudomonas aeruginosa*
52	***Enterococcus faecium*** *, Klebsiella pneumoniae*	*Klebsiella pneumoniae*	***Escherichia coli***

Note

Microorganisms in bold were detected by one diagnostic approach only (either by PCR/ESI‐MS or by SoC) and assessed to be of *added value* or relevant. CoNS: coagulase negative *Staphylococci*.

Samples collected from the same patient are marked with the same index. The dates of their blood collections were as follows:

a 27 July 2016 (id 7); 23 August 2016 (id 8).

b 23 March 2016 (id 48); 6 April 2016 (id 13); 1 June 2016 (id 14).

c 27 August 2016 (id 20); 3 September 2016 (id 21); 4 September 2016 (id 22).

d 16 April 2016 (id 34); 17 April 2016 (id 35).

e 10 April 2016 (id 43); 11 April 2016 (id 44).

## References

[mbo31007-bib-0001] Bacconi, A. , Richmond, G. S. , Baroldi, M. A. , Laffler, T. G. , Blyn, L. B. , Carolan, H. E. , … Sampath, R. (2014). Improved sensitivity for molecular detection of bacterial and *Candida* infections in blood. Journal of Clinical Microbiology, 52(9), 3164–3174. 10.1128/JCM.00801-14 24951806 PMC4313132

[mbo31007-bib-0002] Karrasch, M. , Geraci, J. , Sachse, S. , Rödel, J. , Löffler, B. , Bauer, M. , … Bloos, F. (2018). Early adjustment of antimicrobial therapy after PCR/electrospray ionization mass spectrometry‐based pathogen detection in critically ill patients with suspected sepsis. Clinical Chemistry and Laboratory Medicine, 56(8), e207–e209. 10.1515/cclm-2017-1110 29626413

[mbo31007-bib-0003] Makristathis, A. , Harrison, N. , Ratzinger, F. , Kussmann, M. , Selitsch, B. , & Forstner, C. , … Burgmann, H. (2018). Substantial diagnostic impact of blood culture independent molecular methods in bloodstream infections: Superior performance of PCR/ESI‐MS. Scientific Reports, 8, 1–9. 10.1038/s41598-018-34298-7 30375435 PMC6207717

[mbo31007-bib-0004] O’Dwyer, M. J. , Starczewska, M. H. , Schrenzel, J. , Zacharowski, K. , Ecker, D. J. , Sampath, R. , … Vincent, J.‐L. (2017). The detection of microbial DNA but not cultured bacteria is associated with increased mortality in patients with suspected sepsis—a prospective multi‐centre European observational study. Clinical Microbiology and Infection, 23(3), 208.e1–208.e6. 10.1016/j.cmi.2016.11.010 27890455

[mbo31007-bib-0005] Opota, O. , Jaton, K. , & Greub, G. (2015). Microbial diagnosis of bloodstream infection: Towards molecular diagnosis directly from blood. Clinical Microbiology and Infection, 21(4), 323–331. 10.1016/j.cmi.2015.02.005 25686695

[mbo31007-bib-0006] Özenci, V. , Patel, R. , Ullberg, M. , & Stralin, K. (2018). Demise of PCR/electrospray ionization‐mass spectrometry as an infectious diseases diagnostic tool. Clinical Infectious Diseases, 66(3), 452–455. 10.1093/cid/cix743 29020209

[mbo31007-bib-0007] Peker, N. , Couto, N. , Sinha, B. , & Rossen, J. W. (2018). Diagnosis of bloodstream infections from positive blood cultures and directly from blood samples: Recent developments in molecular approaches. Clinical Microbiology and Infection, 24(9), 944–955. 10.1016/j.cmi.2018.05.007 29787889

[mbo31007-bib-0008] Tassinari, M. , Zannoli, S. , Farabegoli, P. , Pedna, M. F. , Pierro, A. , Mastroianni, A. , … Sambri, V. (2018). Rapid diagnosis of bloodstream infections in the critically ill: Evaluation of the broad‐range PCR/ESI‐MS technology. PLoS ONE, 13(5), 1–12. 10.1371/journal.pone.0197436 PMC5953471 29763469

[mbo31007-bib-0009] Tkadlec, J. , Peckova, M. , Sramkova, L. , Rohn, V. , Jahoda, D. , Raszka, D. , … Drevinek, P. (2019). The use of broad‐range bacterial PCR in the diagnosis of infectious diseases: A prospective cohort study. Clinical Microbiology and Infection, 25(6), 747–752. 10.1016/j.cmi.2018.10.001 30321604

[mbo31007-bib-0010] Vincent, J.‐L. , Brealey, D. , Libert, N. , Abidi, N. E. , O’Dwyer, M. J. , Zacharowski, K. , … Singer, M. (2015). Rapid diagnosis of infection in the Critically ill, a multicenter study of molecular detection in bloodstream infections, pneumonia, and sterile site infections. Critical Care Medicine, 43(11), 2283–2291. 10.1097/CCM.0000000000001249 26327198 PMC4603364

